# Supplementary material for: Unveiling genomic rearrangements in engineered iPSC lines by optical genome mapping
Source: Mol Ther Methods Clin Dev. 2025 Nov 15;33(4):101644. doi: 10.1016/j.omtm.2025.101644 (PMC12701999; doi:10.1016/j.omtm.2025.101644)
Supplement: Document S1. Figures S1–S12 [file mmc1.pdf]

OMTM, Volume *33*

Supplemental information

Unveiling genomic rearrangements in engineered iPSC lines by optical genome mapping

Darren Finlay, Pooja Hor, Benjamin H. Goldenson, Xiao-Hua Li, Rabi Murad, Dan S. Kaufman, and Kristiina Vuori

Table S1. Unique SVs detected. An Excel file of Dual Analysis of each unique SV not present in parental cells detected. Only SVs within 12kbp of a canonical gene are presented for clarity.

A

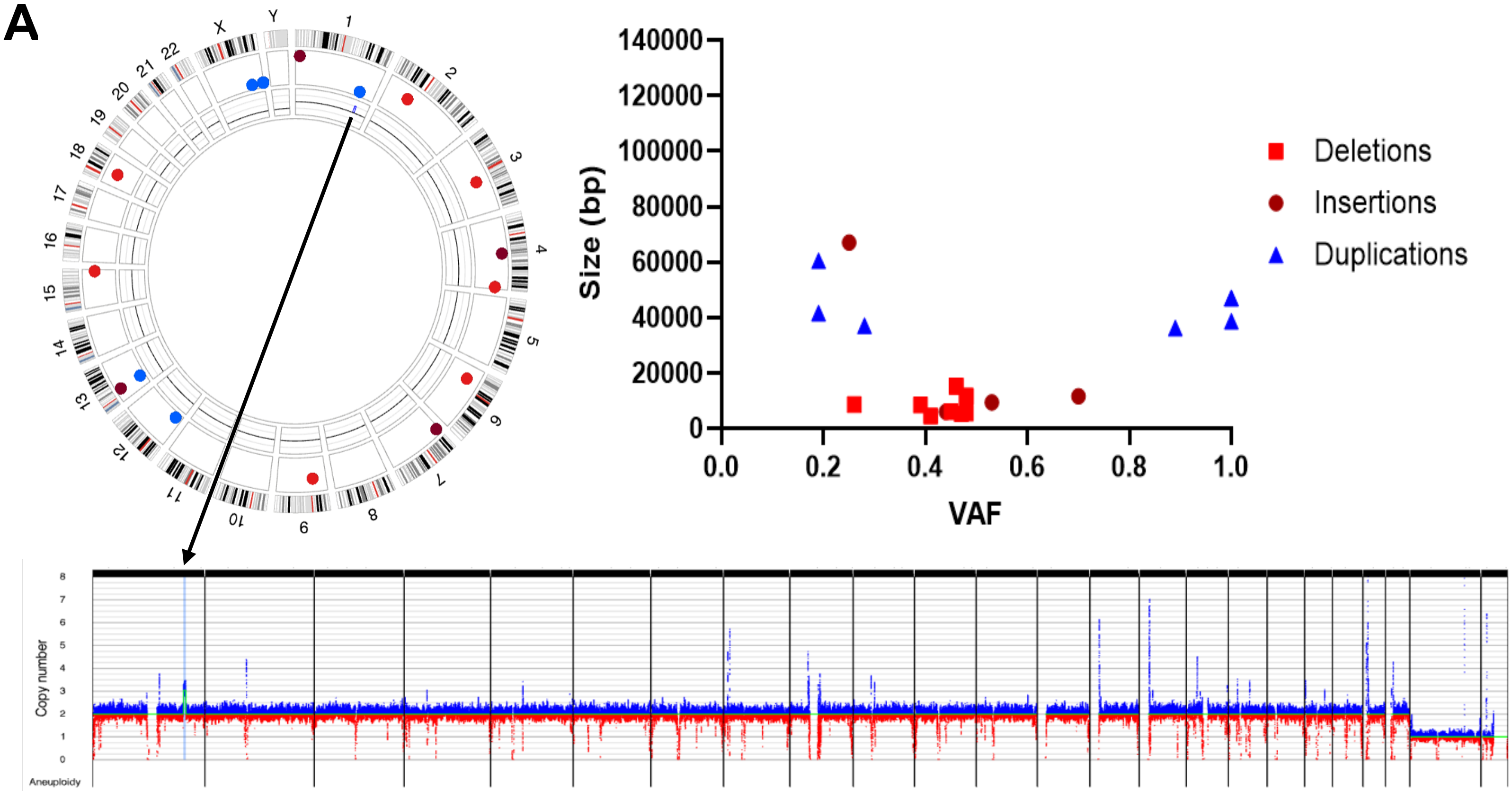

B

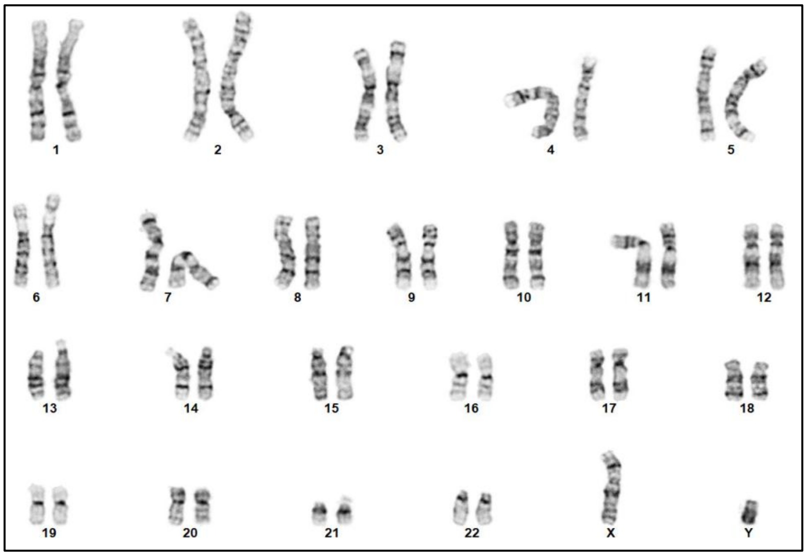

C

WT iPSC (Umbilical)	OGM	Karyotyping
Results	Human Male (46, XY)	46, XY[19]; Normal Human Male Karyotype
Notes	• 8 unique rare deletions noted<br>• Most SVs have VAF ≈ 0.5, indicating heterozygous variants present in all cells.<br>• CNV gain on Chr. 1 observed	• Non-clonal aberration at 46,XY,add(19)(q13.4)[1] is likely a technical artifact

Figure S1. Evaluation of Genetic Fitness in the Parental Wild-Type iPSC Line (Umbilical) Using Rare Variant Analysis (OGM) and Karyotyping.
(A) Circos plot showing whole-genome structural variant (SV) distribution detected by Optical Genome Mapping (OGM).
(B) Representative karyogram of the same iPSC line.
(C) Summary table comparing SVs identified by OGM with conventional karyotype findings in the umbilical cord blood-derived wild-type iPSC line.

A

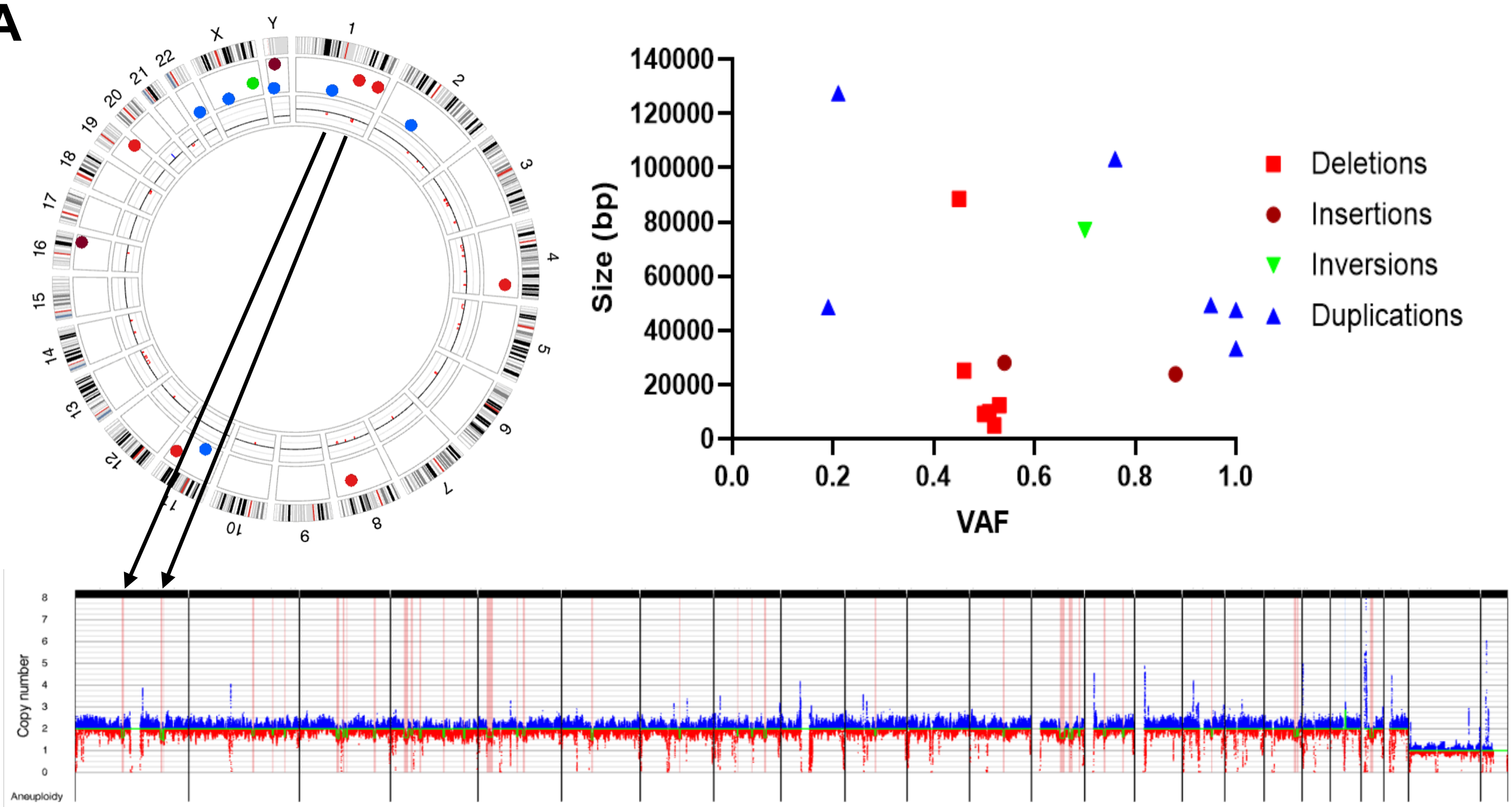

B

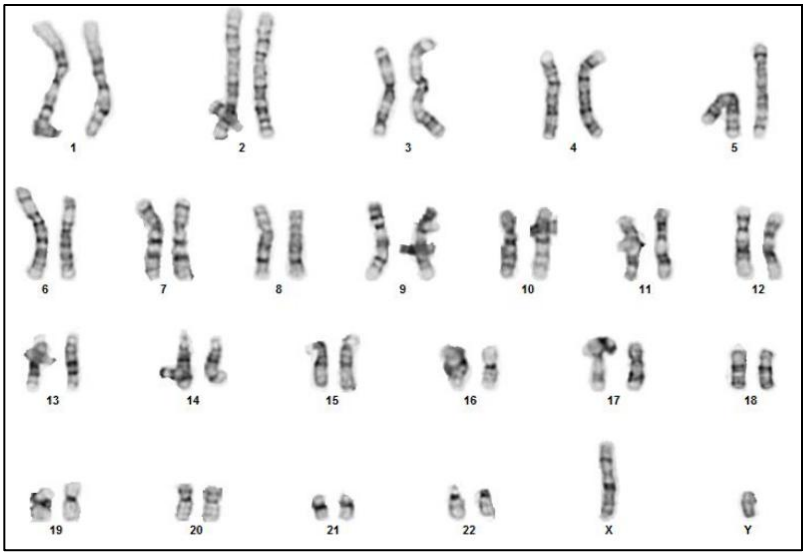

C

WT iPSC (CD34⁺)	OGM	Karyotyping
Results	Human Male (46, XY)	46, XY; Normal Human Male Karyotype
Notes	• 6 unique deletions detected<br>• 2 unique insertions detected<br>• 1 inversion<br>• 6 duplicated regions identified<br>• CNV loss on Chr. 1 observed	

Figure S2. Evaluation of Genetic Fitness in the Parental Wild-Type iPSC Line (CD34$^+$) Using Rare Variant Analysis (OGM) and Karyotyping.
(A) Circos plot showing whole-genome structural variant (SV) distribution detected by Optical Genome Mapping (OGM).
(B) Representative karyogram of the same iPSC line.
(C) Summary table comparing SVs identified by OGM with conventional karyotype findings in the CD34$^+$ cord blood-derived wild-type iPSC line.

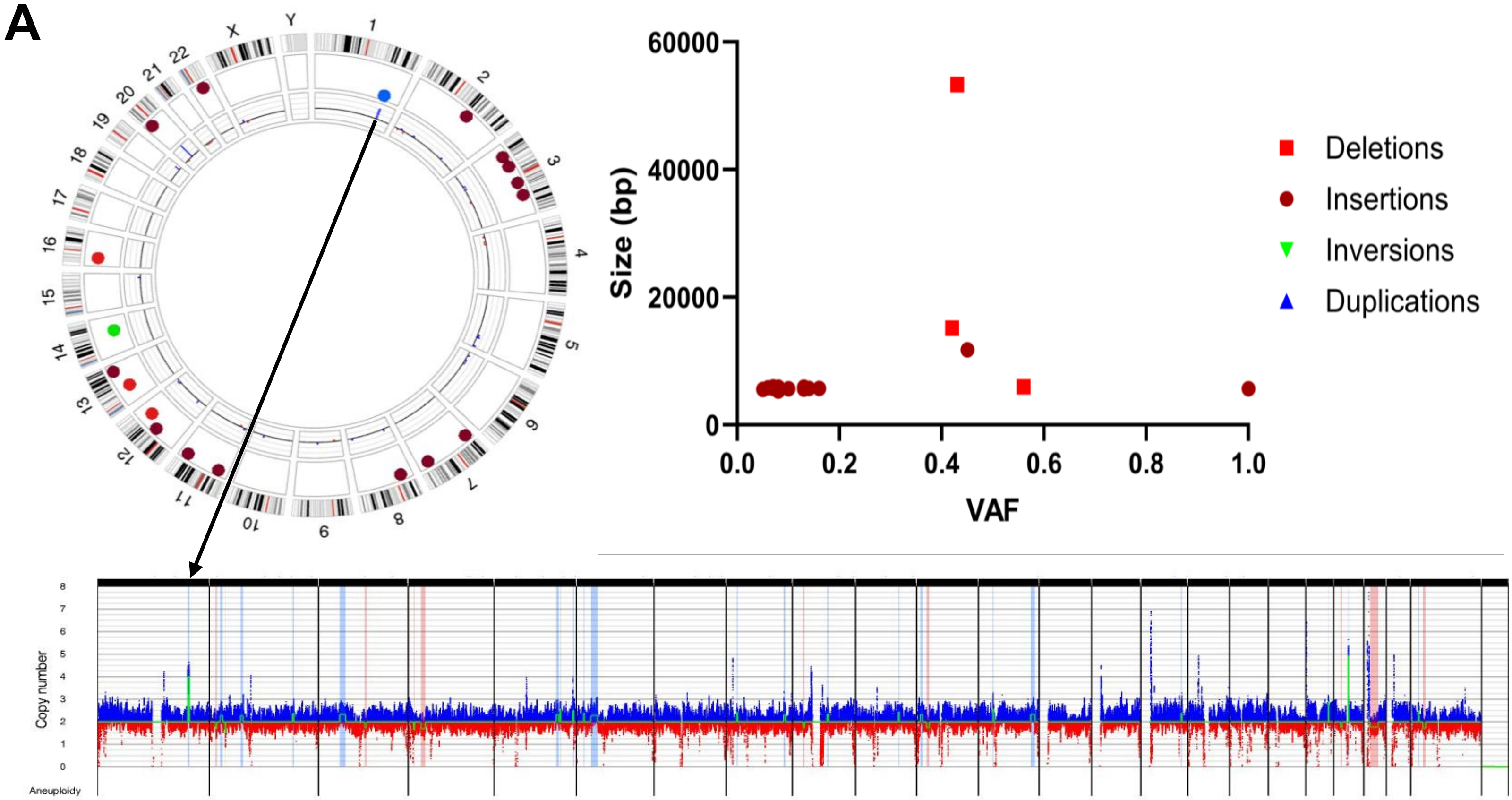

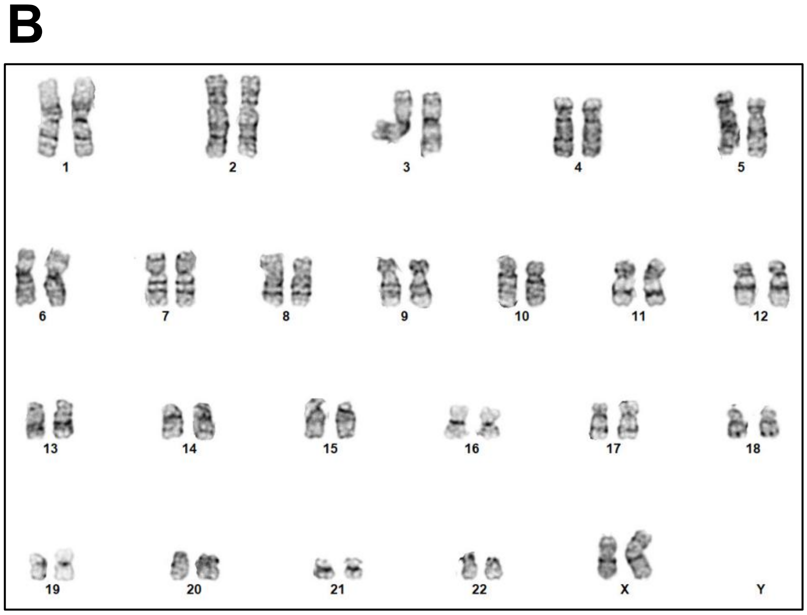

C

CISH-KO iPSC	OGM	Karyotyping
Results	Human Female (46, XX)	46, XX; Normal Human Female Karyotype
Notes	• 3 unique deletions detected<br>• 14 unique insertions detected<br>• 1 inversion (148.4kbp)<br>• CNV gain (3.98Mbp) on Chr.1	

Figure S3. Evaluation of Genetic Fitness in the CISH-KO iPSC Line Using Rare Variant Analysis (OGM) and Karyotyping.
(A) Circos plot showing whole-genome structural variant (SV) distribution detected by Optical Genome Mapping (OGM).
(B) Representative karyogram of the same iPSC line.
(C) Summary table comparing SVs identified by OGM with conventional karyotype findings in the CISH-KO iPSC line.
